# Evolutionary Comparison Provides Evidence for Pathogenicity of *RMRP* Mutations

**DOI:** 10.1371/journal.pgen.0010047

**Published:** 2005-10-21

**Authors:** Luisa Bonafé, Emmanouil T Dermitzakis, Sheila Unger, Cheryl R Greenberg, Belinda A Campos-Xavier, Andreas Zankl, Catherine Ucla, Stylianos E Antonarakis, Andrea Superti-Furga, Alexandre Reymond

**Affiliations:** 1 Division of Molecular Pediatrics, Centre Hospitalier Universitaire Vaudois, Lausanne, Switzerland; 2 Department of Genetic Medicine and Development, University of Geneva Medical School and University Hospitals of Geneva, Geneva, Switzerland; 3 The Wellcome Trust Sanger Institute, Cambridge, United Kingdom; 4 Center for Pediatrics and Adolescent Medicine, Freiburg University Hospital, Freiburg, Germany; 5 Division of Clinical and Metabolic Genetics, Hospital for Sick Children, Toronto, Ontario, Canada; 6 Metabolic Service, Section of Genetics and Metabolism, Health Sciences Centre, Winnipeg, Manitoba, Canada; 7 Center for Integrative Genomics, University of Lausanne, Lausanne, Switzerland; Johns Hopkins Institute, United States of America

## Abstract

Cartilage-hair hypoplasia (CHH) is a pleiotropic disease caused by recessive mutations in the *RMRP* gene that result in a wide spectrum of manifestations including short stature, sparse hair, metaphyseal dysplasia, anemia, immune deficiency, and increased incidence of cancer. Molecular diagnosis of CHH has implications for management, prognosis, follow-up, and genetic counseling of affected patients and their families. We report 20 novel mutations in 36 patients with CHH and describe the associated phenotypic spectrum. Given the high mutational heterogeneity (62 mutations reported to date), the high frequency of variations in the region (eight single nucleotide polymorphisms in and around *RMRP*), and the fact that *RMRP* is not translated into protein, prediction of mutation pathogenicity is difficult. We addressed this issue by a comparative genomic approach and aligned the genomic sequences of *RMRP* gene in the entire class of mammals. We found that putative pathogenic mutations are located in highly conserved nucleotides, whereas polymorphisms are located in non-conserved positions. We conclude that the abundance of variations in this small gene is remarkable and at odds with its high conservation through species; it is unclear whether these variations are caused by a high local mutation rate, a failure of repair mechanisms, or a relaxed selective pressure. The marked diversity of mutations in *RMRP* and the low homozygosity rate in our patient population indicate that CHH is more common than previously estimated, but may go unrecognized because of its variable clinical presentation. Thus, *RMRP* molecular testing may be indicated in individuals with isolated metaphyseal dysplasia, anemia, or immune dysregulation.

## Synopsis

Cartilage-hair hypoplasia is a genetic condition named after two of its most conspicuous features, short bones and sparse hair, but it affects blood-forming tissues, immune system, and intestine. It is caused by sequence mutations in *RMRP,* a small gene that codes for a structural RNA component of an RNAse complex whose biological functions have been elusive so far. The small *RMRP* gene carries a surprisingly high number of sequence variations, and because its transcript is not translated into protein and its function in the cell is still unclear, distinction between harmless variants and disease-causing mutations (more than 60 have been found so far by the authors and others) is difficult. The authors have sequenced the *RMRP* gene in several species covering the whole class of mammals and found that the gene is remarkably conserved between species. Interestingly, mutations occurring in conserved (probably functionally important) regions of the gene appear to be disease-producing, whereas those occurring in regions where evolution is more relaxed seem to be harmless variants. These results will help in counseling affected individuals and their families, and may lead to the discovery of the real function of this mysterious gene.

## Introduction

Cartilage-hair hypoplasia (CHH; MIM#250250) is a pleiotropic disease involving different organs and tissues. Its most frequent clinical manifestations are short-limb dwarfism with metaphyseal dysplasia, joint laxity, fine and sparse hair, anemia, and immune deficiency [1–4], but Hirschsprung disease, impaired spermatogenesis, and malignancies (particularly leukemias, lymphomas, and skin cancer) also occur at increased frequency [5–8].

Mutations in *RMRP,* a gene encoding an RNA component of the mitochondrial RNA processing ribonuclease (RNase MRP) [9], were found to be responsible for CHH [10,11]. Among the known functions of RNase MRP are pre-rRNA processing in the nucleolus and cleavage of RNA primers necessary for DNA replication in the mitochondrion; but it is likely that the RNA molecule coded by *RMRP* has other yet-unidentified functions [12–18]. Forty-two different mutations have been reported so far in 91 Finnish, 47 non-Finnish Caucasian, and two Japanese families [11,19–22]. Given the clinical and radiographic variability of CHH, molecular diagnosis is important both for appropriate counseling and for allowing relevant preventive measures. However, because the *RMRP* RNA is not translated into a protein, it is difficult to evaluate the pathogenicity of a sequence variant. Moreover, the small *RMRP* gene (coding region of 265 base pairs [bp]) has two remarkable features: a very high density of single nucleotide polymorphisms (SNPs) [19,20,22] and an extensive series of allelic, putatively pathogenic variants. For this reason, molecular diagnosis and genetic counseling are particularly difficult.

We report here yet another series of 20 novel *RMRP* mutations in CHH; more importantly, we show that *RMRP* pathogenic mutations, but not SNPs, occur in nucleotides conserved in the entire mammalian class. The implications of these findings in connection with the wide clinical spectrum of CHH are discussed.

## Results

### Mutations and Polymorphisms in CHH Patients

We performed mutation analysis of the *RMRP* gene in 36 patients with suspected CHH. Clinical data are summarized in Table 1. Twelve patients had only skeletal manifestations of the disease during infancy and childhood, 11 patients had also severe immune deficiency, whereas 13 patients had an intermediate phenotype including skeletal dysplasia and at least one extra-skeletal manifestation: recurrent infections and/or subclinical immune deficiency, anemia and/or leucopenia, autoimmune manifestations, hair hypoplasia (defined as thin hair, often lightly colored, and rarely needing a haircut), or Hirschsprung disease.

**Table 1:**
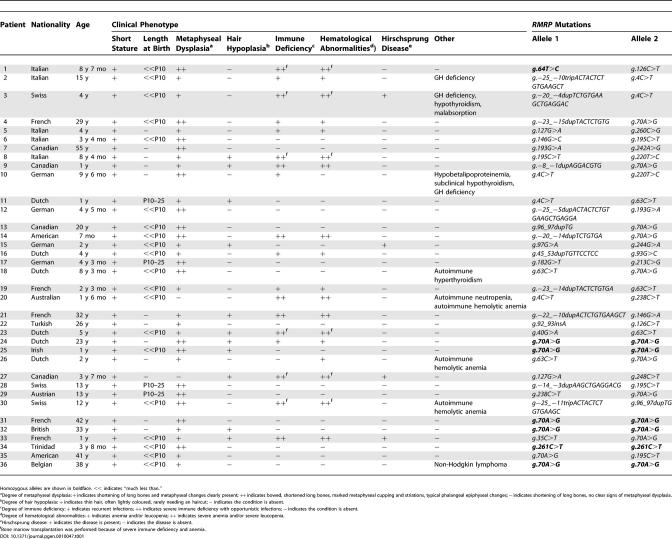
Clinical and Molecular Data of the 36 Studied CHH Patients

Thirty probands were found to be compound heterozygotes for putative pathogenic variants (Table 1), whereas the six remaining probands were true homozygotes for a known pathogenic mutation (see below). We identified 20 previously unreported, putatively pathogenic mutations; five of them are located in the promoter region and 15 in the transcribed region. Table 2 details all *RMRP* mutations reported to date and their localization, as well as the number of reported affected families.

**Table 2:**
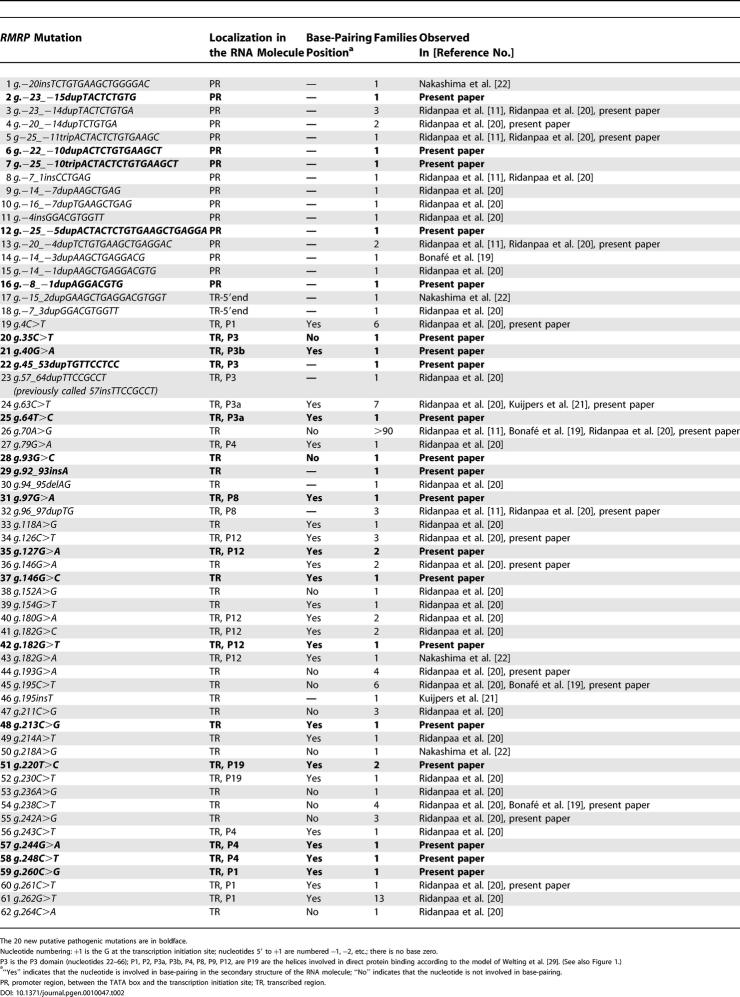
*RMRP* Variants: 20 New Putative Pathogenic Mutations and Review of Reported Mutations

**Figure 1:**
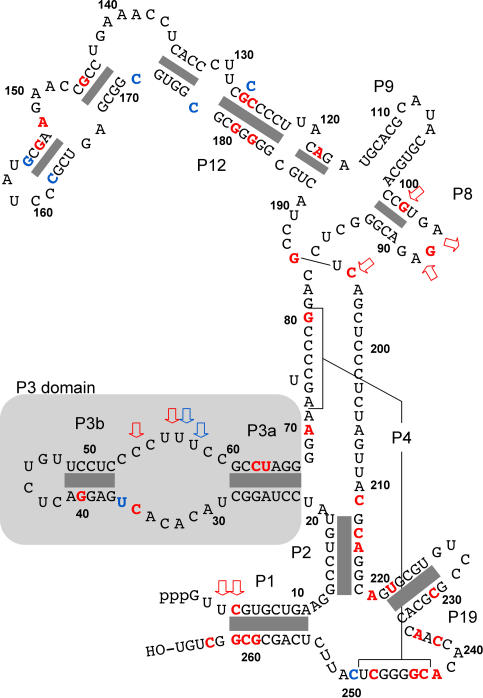
*RMRP* Gene Product The RNA molecule of the human RNase MRP complex is shown according to the model of Welting et al. [29]. Nucleotides affected by mutations (single nucleotide changes) are in red; polymorphisms and rare variants are in blue. Insertion/duplication/deletions are indicated as red (mutations) or blue (putative polymorphisms) arrows. Regions of base-pairing are indicated as gray boxes. The P3 domain (nucleotides 22–66) is shaded.

We identified five new insertions/duplications of 8 to 32 bp localized in the last 25 nucleotides of the promoter region 5′ to the transcription initiation site (Tables 1 and 2). All duplications/insertions in the promoter region result in an increased distance between the TATA box and the transcription initiation site. This type of mutation was always found in compound heterozygosity with a single nucleotide mutation in the transcribed region and never homozygous or in compound heterozygosity with another promoter duplication/insertion.

Among all mutations located within the transcribed region, six are duplication/insertions of 1–17 nucleotides, one is a 2-bp deletion, and the remaining variants are single nucleotide changes (Table 2). Mutations in the transcribed region are located throughout the RNA molecule (Figure 1; Table 2), with no preference in any of the different structural domains. Among the 39 single nucleotide mutations in the transcribed region, 12 are located at nucleotides not involved in base-pairing in the secondary structure of the RNA molecule (Figure 1; Table 2) [18,23,24]. None of the 20 new putative pathogenic mutations reported here were found in 100 non-Finnish Caucasian controls.

The different parental origin of mutations in compound heterozygote patients was confirmed in 23 out of 24 families for which parental DNA was available. In family 27 (Table 1), mutation *g.248C>T* was carried by the father, whereas the *g.127G>A* change was not present in maternal DNA or in that of two unaffected sons. Microsatellite marker analysis of the region around *RMRP* in this family (Figure S1) showed that the same maternal allele is carried by the affected child and by one of the healthy siblings, suggesting that *g.127G>A* is a de novo mutation in this patient, occurring on the maternal allele. However, maternal germinal mosaicism could not be excluded.

Unlike in the Finnish/Amish population, only five patients (24, 25, 31, 32, and 36 in Table 1) were homozygous for the *g.70A>G* CHH mutation. One patient (34 in Table 1) was homozygous for another known pathogenic mutation *(g.261C>T)*. No parental consanguinity was reported in our sample.

We have previously identified seven frequent SNPs within and around *RMRP* and four rare variants (Table 3) [19]. In a Japanese control population (130 alleles), four other rare sequence changes *(g.36T>G, g.55–56insC, g.162C>T,* and *g.172C>T)* have been reported [22]. These variants were found in controls only in heterozygosity and therefore their neutrality is not proven. One more polymorphism has been identified in the present study: *g.127G>C*. This variant was found in 1% of non-Finnish Caucasian controls (two chromosomes out of 200) and observed in one control individual in proven compound heterozygosity with a known pathogenic mutation *(g.70A>G)* (*trans* phase confirmed by parental DNA testing).

**Table 3:**
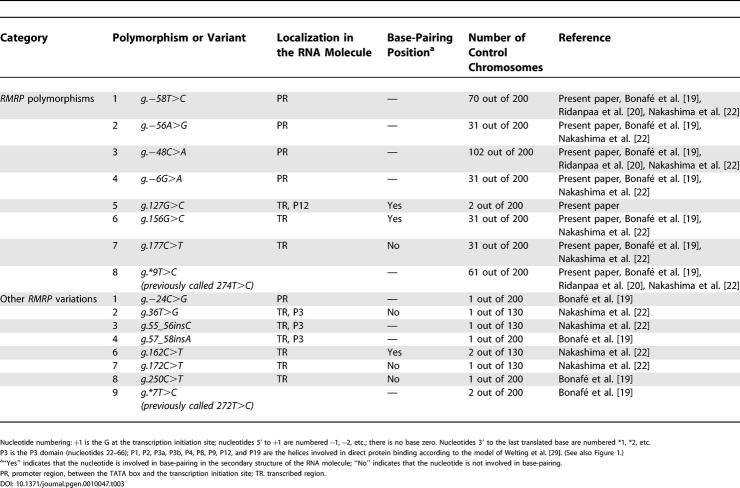
*RMRP* Polymorphisms and Other Rare Variants

Sequence analysis of 100 controls did not detect any putative pathogenic mutation (newly identified and previously described).

### Comparative Genomic Analysis of *RMRP*

**Figure 2:**
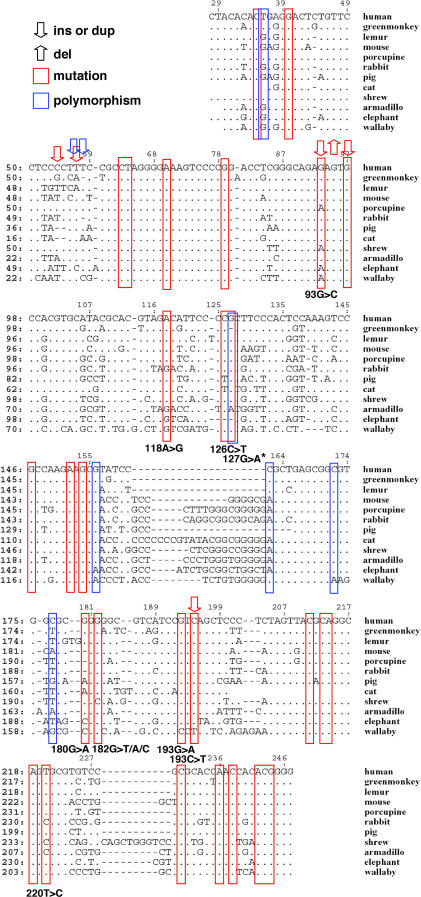
Multiple Sequence Alignment of the *RMRP* Transcribed Region The sequence alignment of 12 mammalian species (11 placental and 1 marsupial) of the *RMRP* transcribed region (from nucleotide g.28 to g.247 of the human sequence) is shown. Putative pathogenic mutations are indicated as red boxes (single nucleotide changes) or red arrows (insertion/duplications/deletions); polymorphisms and rare variants are indicated as blue boxes (single nucleotide changes) or blue arrows (insertion/duplications/deletions). Conservation of nucleotides was analyzed for the single nucleotide substitutions (putative mutations or polymorphisms) included in the alignment interval (from g.28 to g.247). Positions were considered as conserved if 11 out of 12 species had the same nucleotide.

**Figure 3:**
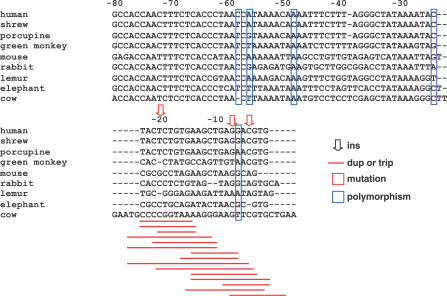
Multiple Sequence Alignment of the *RMRP* Promoter Region The sequence alignment of the *RMRP* promoter region of nine mammalian species (from nucleotide g.−80 to the transcription initiation site of the human sequence) is shown. Polymorphisms and rare variants are indicated as blue boxes (single nucleotide changes); red arrows indicate pathogenic insertions, and red lines indicate pathogenic duplications and triplications. Conservation of nucleotides was analyzed for the single nucleotide substitutions (putative polymorphisms) included in the alignment interval (from g.−80 to g.−1). Positions were considered as conserved if eight out of nine species had the same nucleotide.

To address the significance of sequence changes identified in humans, we compared the human *RMRP* gene against a set of orthologous sequences from mammalian species covering Classes I, II, III, and IV of the Eutherians [25], the Metatherians, and the Monotremes. We successfully PCR amplified and sequenced the *RMRP* transcript regions from genomic DNA of green monkey *(Cercopithecus aethiops)*, ring-tailed lemur *(Lemur catta)*, brush-tailed porcupine *(Atherurus africanus)*, rabbit *(Oryctolagus cuniculus)*, pig *(Sus scrofa)*, cat *(Felis catus)*, white-toothed shrew *(Crocidura russula)*, nine-banded armadillo *(Dasypus novemcinctus)*, African elephant *(Loxodonta africana)*, and tammar wallaby *(Macropus eugenii)* and aligned them to human and mouse *RMRP* (Figure 2). The promoter region of the gene was amplified from white-toothed shrew *(Cr. russula),* brush-tailed porcupine (*A. africanus),* rabbit *(Ory. cuniculus),* green monkey *(Ce. aethiops),* ring-tailed lemur *(Le. catta),* cow *(Bos taurus),* and African elephant *(Lo. africana)* (Figure 3).

The multiple species alignment of the *RMRP* transcribed region showed that 23 putative pathogenic mutations out of 29 are located in strongly conserved nucleotides (same nucleotide in at least 11 out of 12 species) (Figure 2). In contrast, the three polymorphisms located within the coding region (positions g.127, g.156, and g.177) are located in non-conserved nucleotides. Similarly, multiple sequence alignment of the promoter region from −80 to the transcription initiation site showed that only one out of four polymorphisms in this region is located in a conserved position (Figure 3). Three out of four examined rare variants also affect non-conserved nucleotides (g.−24, g.36, and g.162) (Figures 2 and 3).

## Discussion

### *RMRP* Structure and Mutations

The *RMRP* gene encodes a non-translated RNA that forms a cage-shaped structure in the core of the ribonuclease enzymatic complex. Although initially isolated in the mitochondrion, the enzyme is predominantly localized in the nucleolus [26]. Different protein subunits bind to specific domains of the RNA secondary structure [18,24,27–29]. The most recent model of protein–RNA interactions of RNase MRP [29] shows that some regions of the RNA molecule are critical for protein binding (Figure 1). The P3 domain (nucleotides 22–66) is involved in direct binding of several protein subunits (hPop1, hPop4, Rpp20, and Rpp25) [24,27,29]. This region contains also the nucleolar localization signal region (nucleotides 23–62), previously reported as mutation free [20] except for one insertion *(g.57insTTCCGCCT)*, which did not change the signal sequence. We observed three novel mutations *(g.35C>T, g.40G>A,* and *g.45_53dupTGTTCCTCC)* in the nucleolar localization signal region. In the P3 domain mutation, *g.63C>T* is recurrent and was observed in five unrelated families in this study: four of them are of Dutch origin and two of them share the same *[g.63C>T] + [g.70A>G]* genotype (Table 1); furthermore, in all four patients, *g.63C>T* segregates with the Finnish haplotype (data not shown), suggesting a possible common origin of the mutation.

Nucleotide 70 maps just after the end of the P3 domain in a conserved region, which can form, in yeast, a 9-bp duplex with 5.8S rRNA [17], suggesting a possible role in small rRNA processing. The Finnish/Amish founder mutation, *g.70A>G,* was found in 18 out of 72 chromosomes in our series of patients. This frequency (26%) is far lower than the 48% reported for non-Finnish populations [20]. It has been shown that this mutation results from enrichment in an isolate population and is thus not a mutational hotspot [30].

Another recurrent mutation in our series is *g.4C >T,* identified in five of the studied families; it is not associated with a specific haplotype (data not shown), suggesting a multiple origin of the mutation. The other mutations are located throughout the RNA molecule, and no clear genotype-phenotype correlation could be recognized. Phenotypes were different in patients with the same genotype, as previously reported for *g.70A>G* homozygote patients in several Finnish studies [5,11].

Compound heterozygosity for a duplication/insertion in the promoter region and a single nucleotide change in the transcribed region occurs frequently: ten out of 36 families in our sample, and seven out of 44 families in previous reports [20]. Duplication/insertions in the promoter region have never been reported in the homozygous state or in compound heterozygosity with another mutation located within the promoter region, either in our series or in previous works [11,19,20,22]. Because *RMRP* is transcribed by RNA polymerase III [18], the distance between the TATA box and the transcription initiation site is critical for efficient transcription [31]. It has been found that duplications/insertions in this region cause reduced quantity of transcript [11]; the fact that no patient has been found to be homozygous or compound heterozygous for such mutations may indicate that the mutations are phenotypically lethal. Thus, it appears that having drastically reduced amounts of *RMRP* RNA, albeit of normal structure, is more deleterious than having normal amounts of *RMRP* RNA containing subtle structural changes.

Both the abundance of SNPs and the number and diversity of pathogenic mutations in *RMRP* are remarkable and at odds with the high conservation of the *RMRP* gene throughout species. It is unclear at present whether the *RMRP* gene is affected by a particularly high incidence of point mutations, or whether there is reduced efficiency of DNA repairing mechanisms specifically for the *RMRP* gene.

In the background of this mutational heterogeneity, we observed in our patient population a rarity of parental consanguinity and a low incidence of homozygosity for any given mutation. The known inverse relationship between parental consanguinity rate and frequency of a recessive disease suggests that CHH is relatively common among recessive diseases. Precise data on the incidence of CHH outside Finland are lacking, and direct ascertainment is difficult because of the variable clinical expression.

### Comparative Genomics

In molecular diagnosis of CHH, the definition of criteria of pathogenicity for any given mutation is a key issue. Because *RMRP* is not translated into protein, any prediction of the consequences of a nucleotide change is difficult. Correct segregation of mutations in the families and absence of mutations in controls are two commonly used criteria. Other suggested criteria are evolutionary conservation of nucleotides involved in CHH-associated mutations and involvement in base-pairing in the secondary structure model [19]. Several mutations that co-segregate with the disease in multiple unrelated families with CHH do not map in base-pairing positions (Figure 1, Table 2). The best example is *g.70A>G,* the most frequent pathogenic allele worldwide, but 12 other putatively pathogenic mutations are also located in non-paired nucleotides of the RNA molecule. Thus, this criterion appears to be of limited usefulness in defining pathogenicity. Conservation through species, instead, is strongly associated with putative pathogenic mutations. Previously reported multiple sequence alignments [23,32] took into account organisms that were phylogenetically quite distant, and did not examine the promoter region of the gene. We aligned the promoter and transcribed region of *RMRP* from multiple placental and one marsupial mammal. Our analysis shows that putative pathogenic CHH-associated mutations involve strongly conserved nucleotides (23 out of 29). Furthermore, positions corresponding to SNPs are mainly non-conserved, both in the promoter and in the transcribed region (nine out of 11, including rare variants).

We noticed two different changes at position 127, the mutation *g.127G>A,* associated with CHH in two different families, and the polymorphism *g.127G>C,* found in 1% of non-Finnish Caucasian controls. This nucleotide is only partially conserved through species, being 127C in *Xenopus* and 127A in yeast [23,32]. In our alignment, armadillo has 127A whereas all the other placentals and wallaby have 127G. Interestingly, we could document a de novo g.127G>A transition in family 27 (Figure S1), indicating a possible mutational hot spot at this position. Similarly at nucleotide position 182, there are three different *RMRP* mutations in CHH patients, *g.182G>T, g.182G>C,* and *g.182G>A;* the latter was also observed de novo in a Japanese family [22].

Among all *RMRP* variants found in controls (Table 3), eight single nucleotide changes are certainly neutral polymorphisms; the other nine variants were found in controls by us and others only in heterozygosity and are therefore not certainly neutral. Interestingly, the majority of rare variants affect non-conserved nucleotides (Figures 2 and 3), suggesting that they represent rare polymorphisms rather than pathogenic mutations. In conclusion, our observations further validate the role of phylogenetic footprinting in assessing pathogenicity.

### CHH Phenotypic Spectrum

CHH is both pleiotropic, with potential involvement of different organs, and variable in its clinical severity. Skeletal manifestations are characterized by metaphyseal dysplasia of variable degree; extra-skeletal manifestations are sparse hair, anemia, leucopenia, immune deficiency, Hirschsprung disease, and other possible organ involvement (endocrine, autoimmune, and digestive) including malignancies. All patients in our sample were ascertained because of short stature and some degree of skeletal changes. We reviewed personally all available radiographs and selected for those patients who had the typical pattern of phalangeal and metaphyseal striations, the degree of which can be variable. We then separated patients in our sample into three arbitrary groups: approximately 33% (12 out of 36) with short stature and skeletal manifestations only; approximately 30% (11 out of 36) with short stature and severe immune deficiency and/or anemia; and approximately 36% (13 out of 36) with skeletal dysplasia and at least one extra-skeletal manifestation, whose immunological and hematological abnormalities were subclinical or caused only mild clinical symptoms. The severity of skeletal manifestations did not correlate with the severity of extra-skeletal disturbances. Indeed, two patients with severe immune deficiency had short stature but only very mild changes on skeletal survey (Table 1). Thus, the spectrum of CHH phenotypes is wide, ranging from isolated metaphyseal dysplasia to severe immune deficiency associated with short stature of variable degree. If at one end of the spectrum there are several patients with only skeletal manifestations of the disorder (skeletal variant) [19,33], we might also expect, at the opposite end of the spectrum, CHH patients with anemia/leucopenia/immune deficiency and normal stature. Although all patients we had studied were short at the time of diagnosis, not all of them were short at birth, indicating that prenatal growth can be normal. Normal birth length was documented in four patients who later developed clear skeletal signs of the disease and no immunologic abnormalities.

The presence of hair hypoplasia is often associated with other extra-skeletal manifestations: Seven patients out of nine with this characteristic had also immunological anomalies and/or Hirschsprung disease. The absence of hair changes and of hematological/immunological involvement should not exclude CHH from the differential diagnosis of a short child with metaphyseal dysplasia. Short fingers and cone-shaped phalangeal epiphyses are a very sensitive marker for CHH; our data indicate that molecular testing for *RMRP* mutations should be considered in all unclear metaphyseal dysplasias. Long-term follow-up will clarify whether patients with *RMRP* mutations, but only skeletal signs at diagnosis, are at increased risk of malignancy like those with immune deficiency. Indeed, patient 36 (Table 1) had short stature with skeletal dysplasia, but was otherwise healthy until developing non-Hodgkin lymphoma in the fourth decade of life.

Hirschsprung disease was diagnosed in four patients out of 36 (11%), a frequency similar to that reported for Finnish patients (9%) [7]. As previously suggested [7], Hirschsprung disease is associated with severely affected patients (Table 1).

Three patients in our series were reported to have growth hormone (GH) deficiency. Previous studies showed that low IGF-1 levels correlate, in CHH patients, with reduced erythropoiesis [34], but true GH deficiency, defined as insufficient response to GHRH (growth hormone-releasing hormone) stimulation, was never demonstrated [35]; moreover, GH treatment has proven to be of little value in CHH patients [36], as well as in other skeletal dysplasias [37,38]. Because the biochemical demonstration of GH deficiency is difficult and dysregulation of the GH-IGF axis has previously been observed in CHH patients, we would consider these data with caution. In addition, other subclinical endocrine abnormalities seem to occur in CHH patients: Abnormal thyroid function was found in three patients in this study.

The association of hypobetalipoproteinemia and CHH (patient 10 in Table 1) has not been previously described. As clinical symptoms of hypobetalipoproteinemia are often not evident in childhood, it is possible that this abnormality is not frequently tested in CHH patients. However, it is unclear whether hypobetalipoproteinemia is primary in this patient, or secondary to subclinical malabsorption. Some clinical manifestations in this CHH family overlap with Shwachman-Diamond syndrome (SDS; MIM#260400) [39], a disease characterized by pancreatic exocrine insufficiency, hematological dysfunction, and skeletal abnormalities, which is in the differential diagnosis of CHH. Interestingly, the gene causing SDS, *SDBS,* plays a role in RNA metabolism [40], suggesting a possible pathogenetic mechanism common to CHH and SDS. Intestinal malabsorption has been described in CHH [41–43], also in association with celiac disease [44]. Although some instances may be explained by opportunistic bowel infections, it is possible that enteropathy is another rare manifestation of CHH pleiotropism. However this association in single cases may also be caused by chance alone.

Autoimmune disorders were present in four patients in our series. Previous reports suggested an autoimmune mechanism underlying the anemia [45,46] and neutropenia [47] in some CHH patients. There is no evidence of autoimmune anemia in most CHH patients, but it is possible that immune dysregulation favors the development of autoimmunity against other organs and tissues in CHH.

## Materials and Methods

### Sample population.

A total of 59 patients referred to our center for short stature and skeletal changes were included in this study; of these, 40 patients showed the typical pattern of phalangeal epiphyseal changes, metaphyseal cupping and striations of CHH (as judged by two or more of the radiographic experts among us: LB, SU, AZ, and ASF), albeit to a variable degree; and 19 had radiological changes that were considered not typical but still compatible with CHH. All 59 patients were tested for *RMRP* mutations by sequencing of the entire *RMRP* coding sequence as well as promoter region. Twenty-three patients tested negative (all 19 patients in the non-typical group, plus four patients considered to have radiographic changes typical for CHH), and 36 patients had mutations in *RMRP*. The patient material was referred to us for non-commercial diagnostic help and accepted under the research terms specified in the European Skeletal Dysplasia Network program (http://www.esdn.org/) (for European patients) or analogously, for non-European subjects. Appropriate informed consent was obtained by the physicians in charge. The clinical data and the radiographic features of each patient were evaluated before molecular testing. The clinical data of the 36 unrelated patients with CHH are summarized in Table 1.

Parental DNA was studied in 24 families. For the remaining 12 patients (patients 2, 7, 8 [adopted], 11, 21, 24 [homozygote], 25 [homozygote], 26, 31 [homozygote], 32 [homozygote], 35, and 36 [homozygote]), parental DNA was not available.

### Molecular analysis of *RMRP* gene.

Genomic DNA was extracted from blood leukocytes according to standard methods; in one patient (patient 5) genomic DNA was extracted from fibroblasts because bone marrow transplantation had been performed before the study.

Genomic DNA of 100 non-Finnish Caucasian control individuals (German, Italian, British, French, Swiss, Australian, and Canadian) has been studied to test for the presence or absence of all variants, including new putative pathogenic mutations, previously reported mutations, and putative polymorphisms.

The coding and promoter regions of the *RMRP* gene were amplified from genomic DNA by PCR and analyzed by fluorescent bidirectional direct sequencing as previously described [19]. Each variant was confirmed by reamplification and resequencing. *Trans* phase of mutations in 24 compound heterozygote patients was verified by confirming the presence of mutations in parental DNA .

Microsatellite marker analysis of the region around *RMRP* in family 27 was performed with the ABI Prism Linkage Mapping Set version 2.5 kit and protocol, in an ABI Prism 3100Avant automatic sequencer (Applied Biosystems, Foster City, California, United States).

### Comparative genomics.

Tissues or genomic DNA from the following species were obtained from different sources (for details see Table S3 of Dermitzakis et al. [48]): *S. scrofa, F. catus, My. myotis, Cr. russula, A. africanus, Ory. cuniculus, Ce. aethiops, Le. catta, D. novemcinctus, Lo. africana, Ma. eugenii, Orn. anatinus, B. taurus**.*** Pairs of oligonucleotides were designed on human DNA sequences in highly conserved regions between human and mouse to amplify either the promoter (5′-GCCACCAACTTTCTCACC-3′ and 5′-GGGACTTTCCCCTAGGC-3′) or the transcribed region (5′-GGCCTGTATCCTAGGCTACA-3′ and 5′-AGCCGCGCTGAGAATGAG-3′) of the *RMRP* gene.

Conservation of single nucleotide substitutions was analyzed on the multiple sequence alignments of the transcribed region (Figure 2) and the promoter region from g.–80 to g.−1 (Figure 3), based on the number of species harboring the same nucleotide. We considered as conserved the positions where at least 11 out of 12 species (in the transcribed region) or eight out of nine species (in the promoter region) had the same nucleotide.

## Supporting Information

Figure S1 Microsatellite Marker Analysis of the Region around RMRP in Family 27 The same maternal haplotype is carried by the affected child and by one of the healthy siblings. Neither the mother nor the unaffected sibling carry g.127G>A, suggesting that this mutation occurred de novo in the affected individual (see also Table 1).
(378 KB PDF)

### Accession Numbers

The GenBank (http://www.ncbi.nlm.nih.gov/Genbank) accession numbers for the PCR-amplified *RMRP* sequences are CC935687, CC935890, CC935995, CC936164, CC936319, CC936439, CC936561, CC936652, and CC936711; the GenBank accession number for the *RMRP* gene is M29916.

## Abbreviations

bp: base pair(s)
CHH: cartilage-hair hypoplasia
GH: growth hormone
SDS: Schwachman-Diamond syndrome
SNP: single nucleotide polymorphism
