# Supplementary material for: Evolutionary Comparison Provides Evidence for Pathogenicity of RMRP Mutations
Source: PLoS Genet. 2005 Oct 21;1(4):e47. doi: 10.1371/journal.pgen.0010047 (PMC1262189; doi:10.1371/journal.pgen.0010047)
Supplement: Supplementary file 1 [file pgen.0010047.sg001.pdf]

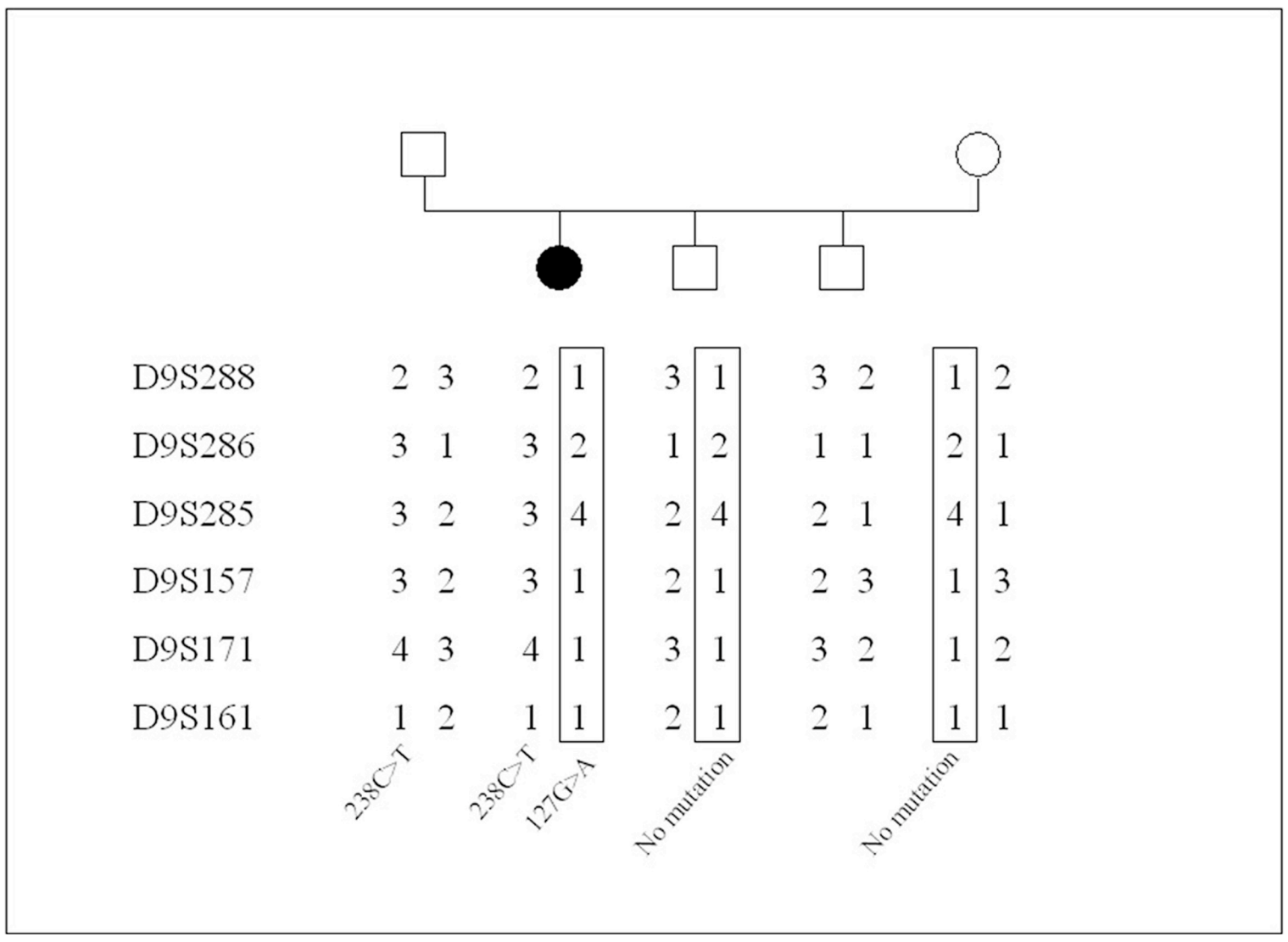
D9S288 2 3 2 1 3 1 3 2 1 2
D9S286 3 1 3 2 1 2 1 1 2 1
D9S285 3 2 3 4 2 4 2 1 4 1
D9S157 3 2 3 1 2 1 2 3 1 3
D9S171 4 3 4 1 3 1 3 2 1 2
D9S161 1 2 1 1 2 1 2 1 1 1
238C>T
238C>T
127G>A
No mutation
No mutation
